# Migraine, Allergy, and Histamine: Is There a Link?

**DOI:** 10.3390/jcm12103566

**Published:** 2023-05-19

**Authors:** Alessandro Ferretti, Mattia Gatto, Margherita Velardi, Giovanni Di Nardo, Thomas Foiadelli, Gianluca Terrin, Manuela Cecili, Umberto Raucci, Massimiliano Valeriani, Pasquale Parisi

**Affiliations:** 1 Pediatrics Unit, Neuroscience, Mental Health and Sense Organs (NESMOS) Department, Faculty of Medicine and Psychology, Sapienza University of Rome, 00189 Rome, Italy; 2 Child Neurology and Psychiatry Unit, Systems Medicine Department, Tor Vergata University of Rome, 00133 Rome, Italy; 3 General and Emergency Department, Bambino Gesù Children’s Hospital, Istituto di Ricerca e Cura a Carattere Scientifico, 00165 Rome, Italy; 4 Pediatric Clinic, Fondazione IRCCS Policlinico San Matteo, 27100 Pavia, Italy; 5 Department of Mother and Child, Gynecological and Urological Sciences, Faculty of Medicine and Dentistry, Sapienza University of Rome, 00185 Rome, Italy; 6 Developmental Neurology Unit, Bambino Gesù Children’s Hospital, Istituto di Ricerca e Cura a Carattere Scientifico, 00165 Rome, Italy

**Keywords:** migraine, allergy, allergic disorders, histamine, antihistamine drugs, histaminergic system

## Abstract

The relationship between migraines and allergies is controversial. Though they are epidemiologically linked, the underlying pathophysiological connection between them remains unclear. Migraines and allergic disorders have various underlying genetic and biological causes. As per the literature, these conditions are epidemiologically linked, and some common pathophysiological pathways have been hypothesized. The histaminergic system may be the clue to understanding the correlation among these diseases. As a neurotransmitter in the central nervous system with a vasodilatory effect, histamine has a well-documented influence on the allergic response and could be involved in the pathophysiology of migraines. Histamine may influence hypothalamic activity, which may play a major role in migraines or may simply influence their severity. In both cases, antihistamine drugs could prove useful. This review examines whether the histaminergic system, particularly H3 and H4 receptors, may provide a mechanistic link between the pathophysiology of migraines and allergic disorders, two common and debilitating conditions. Identifying their connection could help identify novel therapeutic strategies.

## 1. Introduction

Allergists and neurologists regularly receive patients, both pediatric and adult, who suffer from migraines and allergic disorders. However, it is still unclear whether the two disorders are closely related, whether they are triggered by common factors but arise independently, or whether they occur as comorbidities [1]. 

Migraines and allergic disorders are among the most prevalent disorders worldwide [2,3,4,5,6,7,8,9,10]. Therefore, they can occur in the same patient as comorbidities without being directly related. Their prevalence is 15.2% [11,12] and up to 50% [13] for migraine and allergic rhinitis (AR), respectively. No data are currently available on the overall prevalence of allergic disorders (including AR, rhinosinusitis, rhino-conjunctivitis, asthma, atopic dermatitis, eczema, and food allergy). However, the overall prevalence appears to have been increasing over the last few decades [14,15]. In addition, there are no data on the prevalence of atopy, characterized by an excessive immunoglobulin E (IgE) reaction after exposure to allergic triggers in genetically predisposed individuals [16]. Despite their high prevalence, the two disorders have a bidirectional epidemiological relationship, as several population-based studies have shown [17,18,19,20,21,22,23,24,25,26,27,28,29,30,31,32,33,34,35,36,37,38,39,40,41,42,43]. The proportion of patients in which the two diagnoses overlap is not well investigated [29,37,38]. In addition, allergists, otolaryngologists, and neurologists often have patients reporting “sinus headaches”, which is a common, though nonspecific, diagnosis that should be considered an archaic definition [44,45]. Several studies have confirmed that at least 50% of the patients complaining of sinus headaches meet the international criteria for a migraine diagnosis [46,47,48,49]. Although an epidemiological overlap between the two disorders has been identified, the exact link remains unclear. One possible link is that both diseases have a pathophysiological mechanism based on inflammation and immune dysfunction. It is known that atopy, inflammatory mediators, and elevated neuropeptide mediators all contribute to migraines and allergic disorders such as asthma [50,51,52,53,54]. The histaminergic system could also indicate a link between these diseases. A role of histamine in the etiopathogenesis of migraines has been hypothesized due to its modulation of hypothalamic functions [55]. Knowledge of the histaminergic system could improve the diagnostic process and treatment [56,57]. An in-depth understanding of the role of the histaminergic system in the pathogenesis of the two diseases could help identify more effective therapies and ultimately improve the quality of life of patients affected by both disorders. The objective of the present review is to investigate the epidemiological association between migraines and allergic disorders, focusing on their shared biochemical pathways and underlying mechanisms. The hypothesis that histamine and the histaminergic system play a common role in the pathogenesis of both disorders is discussed, as are the potential therapeutic implications.

## 2. Materials and Methods

Electronic databases such as PubMed, Scopus, and Web of Science were searched to investigate the relationship/comorbidity of migraine headaches in subjects with allergic/atopic disorders and vice versa from 2000, as well as studies reporting the role of histamine and the histaminergic system in both these conditions and the role of antihistamine drugs from 1960, using the search terms “headache” and “migraine” with the following prefixes: “allergic disorders”, “atopic disorders”, “allergic rhinitis”, “rhinosinusitis”, “rhino-conjunctivitis”, “asthma”, “atopic dermatitis”, “eczema”, “physiopathology”, “histamine”, “histaminergic system”, and “antihistamine drugs”. Moreover, the reference lists of the articles considered were screened to identify additional publications and cases that satisfied the search criteria. In this review, case series or case reports, observational studies (prospective and retrospective cohorts, case–control, and cross-sectional), original research studies, and pooled analyses of original data were included, while other article types, such as commentaries, letters to the editor, and non-English articles, were excluded. Using a data collection form, the following information was extracted: the first author’s name, publication year, study design, sample size, demographic characteristics such as age and sex, country of the population studied, criteria for enrollment, ascertainments for allergic disorders, and definitions of migraine. Two authors (A.F. and M.G.) extracted the data of interest from the studies.

## 3. Associations between Migraines and Allergic Disorders: Evidence from Population-Based Studies

In the past, the relationship between migraines and allergic/atopic disorders has been a focus of interest [58], even though the reports appear to be conflicting. Population-based studies with large samples and different age groups and countries have been conducted to clarify the epidemiological bidirectional relationship between these disorders [17,18,19,20,21,22,23,24,25,26,27,28,29,30,31,32,33,34,35,36,37,38,39,40,41,42,43,59,60]. The main aim of these was to verify a greater comorbidity of allergic disorders in patients suffering from migraines and vice versa. Other aims were to identify whether allergic disorders are correlated with different types of headaches [20,29], whether family allergy and atopy are negative predictors of migraines [21,38], and whether allergy/atopy severity is correlated with the severity of the migraine attack or its recurrence and vice versa [25,29,34,39,41]. Table 1 summarizes the main results of the prospective and retrospective population-based studies conducted since 2000.

As shown in Table 1, most studies reported that allergic/atopic disorders (including allergic rhinitis and atopic dermatitis), asthma, and migraine are reciprocally significantly associated [17,18,19,20,21,22,23,24,25,26,27]. The overlap between allergic disorders without asthma [37,38,39] and atopic dermatitis/eczema [40,41,42,43] with migraine is also consolidated. Among the allergic disorders, the prevalence of asthma in migraine patients and migraines in asthma patients has been investigated the most, and the two conditions have been found to be epidemiologically associated [28,29,30,31,32,33,34,35,36], except in two retrospective studies [59,60]. In two questionnaire-based cross-sectional surveys, Ronchetti et al. found that headaches were not significantly associated with asthma [59]. Becker et al. observed that the risk of developing asthma was not considerably increased for patients with a general practitioner-recorded migraine diagnosis [60]. Beyond the epidemiological overlap between these conditions, studies have identified other linkages: (1) Among the different types of headaches, migraines are more closely correlated with allergic disorders than tension-type headaches [20,22], and migraines with aura are more closely related to allergic disorders than migraines without aura [20]. (2) A family history of migraines may lead to comorbidity with allergy [38], whereas allergy, migraine, and a parental history of asthma were found to be independent risk factors for the presence of migraine in asthmatics. (3) Allergy/atopy and asthma severity are correlated with the severity and frequency of migraine attacks [29], with the highest risk among asthmatic patients with the greatest number of respiratory symptoms [34]. Lower “degrees of atopy” were associated with less frequent and disabling migraines in younger people, while higher degrees were associated with more frequent migraines in patients with allergic rhinitis; in this regard, the administration of immunotherapy is associated with a decreased prevalence and frequency of and disability arising from migraines in younger people [39]. In addition, severe allergic skin symptoms, such as eczema, accompanied by atopy, fatigue, and sleep disturbances increase the severity of headaches [41]. (4) Childhood allergic diseases, such as asthma, atopic dermatitis, allergic conjunctivitis, and rhinitis [24,26], were found to be associated with migraine headaches in adolescence. In addition to population-based studies, several systematic reviews and meta-analyses (not included in Table 1) have confirmed an association between asthma and migraine [61,62,63]. In a systematic review involving 389,573 participants performed to assess the overlapping risk of migraines in asthma patients and vice versa, the odds ratio (OR) of migraines in asthma patients compared with non-asthmatic individuals was found to be 1.62 (95% CI: 1.43–1.82) and migraines were associated with a significantly increased risk of asthma (relative risk (RR): 1.56; 95% CI: 1.51–1.60; *p* < 0.00001) [61]. A meta-analysis of 15 published population-based studies covering a total of 1,188,780 individuals showed that migraines were associated with increased odds (OR = 1.54; 95% CI: 1.34~1.77) and risk of asthma (HR = 1.42; 95% CI: 1.26~1.60) and asthma was associated with increased odds (OR = 1.45; 95% CI: 1.22~1.72) and risk of migraines (HR = 1.47; 95% CI: 1.41~1.52) [63]. In a systematic review and meta-analysis of observational studies, including seven studies with a total of 549,534 individuals, to determine the association between asthma and migraine, asthma was also associated with increased odds (OR = 1.85; 95% CI: 1.39–2.45) and risk of migraines (RR = 1.70; 95% CI: 1.52–1.90) [62]. However, the same authors concluded that evidence associating asthma and migraine is limited [62]. Population-based studies and derived meta-analyses identify a strong association between migraines and allergic disorders; however, these data are not supported by incontrovertible etiopathogenetic links.

## 4. Hypothesis of a Common Pathophysiological Pathway 

Researchers widely discuss migraines and allergic disorders due to their complex mechanisms that lead to injuries and comorbidities and because they could share common pathophysiological pathways. Some of the most developed theories are listed below.

### 4.1. Immunological Involvement

Several data support an underlying immunological imbalance in both disorders [20,64]. Serum IgG antibodies to 108 common foods were significantly higher in migraineurs than in the control group [65]. The elimination diet significantly reduced the number of headache days and attacks [65,66]. Despite such evidence, the concept of IgG-mediated food allergy and migraines is considered highly controversial [67]. Duodenal biopsy studies do not support a food allergy–migraine association [68]. Serum IgE and histamine levels were significantly higher in migraineurs than in controls, and all migraine patients had significantly higher histamine levels during headaches than during their non-headache intervals [56]. A meta-analysis also reported elevated IgE levels in people with atopic migraine; however, this was not found in those without allergy [53]. The authors found decreased lymphocyte phagocytic function between migraine attacks [53]. However, the same authors concluded that there was no evidence of immune suppression in migraineurs [53].

### 4.2. Environmental Factors and Other Triggers

Both disorders are explained by the interaction of genetic and environmental factors [69,70]. Some environmental triggers, such as weather changes and seasonal variation, foods, exercise, and emotional stress, may trigger both allergic disorders and migraines [47,70,71,72,73]. The Saharan dust with the atmospheric airstream, already associated with asthma and allergic rhinitis [74], has been documented to be able to trigger headaches in rats, inducing c-fos expression in nociceptive neurons within trigeminal nucleus caudalis [75]. Sleep pattern changes may also trigger migraines [76]. In this regard, some allergic disorders, such as atopic dermatitis and eczema, may be responsible for sleep disturbances, increasing the frequency and severity of migraine attacks [22,63,77,78,79]. Increased nasal congestion due to seasonal allergies, in addition to altering the quality of sleep, can irritate the trigeminal nerve in the nose, which could trigger a migraine attack. Nasal congestion and rhinorrhea during a migraine attack were reported based on objective endoscopic evidence [77].

### 4.3. Involvement of Vasoactive Mediators 

Studies in adults suggest that primary headaches could be the first sign of atherosclerosis and platelet aggregation [80,81], and in this regard, P2Y12 platelet inhibitors may have a primary prophylactic role in migraine patients [81]. Platelets are activated during an asthma attack and following an antigen provocation, supporting the hypothesis that platelets may also contribute to the pathogenesis of asthma [82]. Vasoactive intestinal peptide (VIP), a vasoactive mediator, present in the neuroendocrine cells of the airway epithelium, is released along with other neuropeptides and neurotransmitters following exposure to the allergen in asthma [83]. VIP also leads to a marked dilation of cranial arteries; however, it does not trigger migraine attacks in migraineurs, as demonstrated in a randomized controlled trial [84]. These data provide some evidence against a purely vascular origin of migraines, as the authors themselves have commented [84].

### 4.4. Inflammation

The activation of inflammatory mediators (inflammatory interleukins, visinin-like protein 1, YKL-40, lipocalin-2, and TNF-α) may play a role in the pathogenesis of migraines [50,51,52,53,54]. Allergies cause inflammation, which could increase the frequency of migraines. The relationship between migraine and bronchial hyper-reactivity suggests that inflammation is the underlying mechanism in migraine [85]. Transient receptor potential vanilloid subfamily member 1 (TRPV1), expressed in the membranes of sensory afferent fibers and on the trigeminal nociceptors, has been shown to play a key role in the pathophysiology of both migraines and asthma [78,79]. It can be activated by various chemical substances, such as acids, and by heat. Once activated, TRPV1 releases neuropeptides, causing neurogenic inflammation and exerting a vasodilatory effect, which are crucial factors in the generation of migraines [86]. Similarly, TRPV1 expressed in airway C-fiber afferent neurons can be activated by endogenous activators or inhaled irritants, a phenomenon observed in patients presenting with asthma [87].

### 4.5. Parasympathetic and Trigeminal Nerve Involvement

Some authors believe that, in patients affected by rhinosinusitis and migraines, there is a “physiologically coordinated” regulation of trigeminal and parasympathetic nerve activity [88]. Asthma and migraines are linked by parasympathetic hyperactivity [89]. An increased cholinergic tone has been suggested to trigger bronchospasm in asthma [90], and acetylcholine released by parasympathetic afferents can directly activate the trigeminal pain pathway or provoke the degranulation of mast cells in meninges between migraine attacks [91]. Furthermore, calcitonin gene-related peptide (CGRP), secreted by trigeminal nerves, and vasoactive intestinal peptide (VIP), secreted by parasympathetic nerves, could be involved in the pathophysiology of migraines and rhinosinusitis [88].

## 5. Role of the Histaminergic System in Migraines

Studies conducted in recent decades have focused on the role of histamine in the central nervous system (CNS) (regulation of the sleep–wake rhythm, hormonal regulation, and cognitive functions) [92,93]. The effects of neuronal histamine are mediated via G-protein-coupled H1–H4 receptors [94]. H1 and H2 receptors function by modulating vascular permeability [55,95]. H3 and H4 receptors, together with the mast cell system, play a role in nociception [96,97,98,99]. The histaminergic system may play an important role in migraine pathophysiology [56,100], and dietary histamine, similar to other chemical dietary triggers, may play a role in some individuals suffering from migraines [101,102]. The following paragraphs will analyze these aspects.

### 5.1. Histamine and the Central Nervous System

Previous studies have proved that the continuous intravenous infusion of histamine could elicit a headache [103]. This effect is consistent with the hypothesis of mast cells, immunoglobulin E, histamine, and cytokines [94] playing mediating roles in migraine pathways. Histaminergic fibers are represented in the migraine-related neural structures [100], and several studies have documented higher levels of histamine and IgE in patients suffering from migraines compared with controls [56,104]. Histamine, a diamine linked to gastric secretion, mast cells, and the immune system [94], has an important function as a neurotransmitter in the CNS [92]. Histaminergic neurons originate from the tuberomammillary nucleus (TMN) of the posterior hypothalamus [100], which projects to a suprachiasmatic nucleus (SCN) [105,106] of the hypothalamus itself. The histamine released from the TMN helps to modulate the circadian rhythm, the sleep–wake cycle, pituitary hormone secretion, thermoregulation, energy expenditure, and food intake [94,107]. During wakefulness, its activation is variable; however, during somnolence and sleep, it is no longer activated through gamma-aminobutyric acid connections [108]. The extracellular levels of histamine in the preoptic/anterior hypothalamus follow the oscillations of the different sleep phases (wake > non-REM sleep > REM sleep) [108], providing further evidence of the importance of histamine in circadian regulation [55]. The close correlation between the regulation of circadian rhythms and migraines [109] has also been confirmed through migraine mouse model studies, which have suggested an inadequate coping mechanism compared to that of the general population as a possible cause [110]. The pain response and modulation of headache attacks are other histamine-mediated hypothalamic functions [55,111]. Rat studies have demonstrated strict connections between the TMN, thalamic nuclei, and the trigeminal output, indicating that histamine may trigger headache stressors involved in the perception of whole-body allodynia (abnormal skin sensitivity) and photophobia (abnormal sensitivity to light) during migraine [112,113]. The histaminergic system is also connected with the trigeminal vascular system, known to be involved in migraine pathophysiology [114,115,116], and with orexinergic, noradrenergic, and serotonergic areas [55,92]. For these reasons, it plays a role in a wide range of cognitive functions and acts as a general index of stress [117]. Histamine is released in response to stressors that could elicit or worsen migraine attacks in genetically predisposed individuals [118]. Dietary histamine, similar to other chemical food triggers, might play a role in some individuals suffering from migraines. Some foods and alcoholic beverages that are high in histamine could trigger or worsen migraine attacks in these people due to histamine’s action on the CNS [101,102]. Symptoms may be worse in individuals with histamine intolerance due to impaired histamine degradation based on reduced activity of diamine oxidase, the main enzyme involved in the metabolism of ingested histamine [119,120].

### 5.2. H1 and H2 Receptors

H1 receptor (H1R) seems to be linked to the control mechanisms of the tight junctions of the BBB. In cultured brain endothelial cells, the histaminergic system increases synaptic activity by modulating glucose intake [121]. When H1R is stimulated, it modulates vascular permeability by inducing the production of nitric oxide (NO) by activating nitric oxide synthase [55]. Studies of glyceryl trinitrate (GTN) and histamine-induced headaches have indicated that NO may initiate migraine attacks [95]. The substances that have been shown to reliably cause more headaches than placebos in single-dose experiments were GTN and histamine, with NO as the common mediator [95]. Histamine 2 receptor (H2R), abundant in the basal ganglia, amygdala, and hippocampus and commonly residing close to H1R [100], also acts in hippocampal long-term potentiation, memory consolidation, and spatial navigation and regulates vascular tone and BBB permeability [100,122]. Finally, H2R plays a role in the expression of vascular protective factors in astrocytes and brain microvascular endothelial cells [123].

### 5.3. H3 and H4 Receptors

The H3 receptor (H3R) is located presynaptically (in peripheral and CNS) and inhibits neurons, leading to the auto-inhibition of the histaminergic neurons themselves [55]. Some polymorphisms associated with impaired H3R functioning have been associated with a greater risk for the development of some neurological diseases, including migraines [124,125]. These findings can be explained by the anti-nociceptive function of these receptors [96,97,98], a function possibly related to a similar action in the modulation of the release of CGRP and pain substance (SP) through the prejunctional histamine H3R located on the peripheral endings of the sensory nerves [113]. Moreover, histamine has a selective affinity for H3R and may specifically inhibit the neurogenic edema response involved in migraine pathophysiology, modulating the vascular permeability [126,127]. The H4 receptor (H4R) shows considerable homology with the H3R (35%) and is primarily found in peripheral tissues and immune cells [55]. Recently, a functional expression of H4R within neurons of the CNS in the rat lumbar dorsal root ganglia (DRG) and in the lumbar spinal cord [100,128] has been reported [129,130]. H4R modulators may play a role in pain modulation (antinociceptive effect) as well as inflammatory and immune processes, as suggested by animal studies [128,129].

### 5.4. Mast Cells and Pain Neuromodulation

Mast cells may represent another tight link between the histaminergic system and the modulation of nociception associated with migraines [55,100,131]. The proximity of mast cells to nociceptive nerve endings in many cutaneous and deep tissues, including meninges, suggests a functional neuroimmune interaction involving mast cells and nociceptive neurons [52,132]. Migraine is generally accepted to be mediated by the prolonged activation of meningeal nociceptors [131,133]. In rat studies on meningeal nociceptors, the degranulation of dural mast cells induced a prolonged state of excitation in meningeal nociceptors. Nociceptor interaction was associated with an increased expression of nociception-associated kinases (pERK) and with a downstream activation of the nucleus of the spinal trigeminal, indicated by the increased expression of c-fos [133]. In addition, activated macrophages, microglia, and mast cells in the CNS release pro-inflammatory cytokines, which cause an increase in the arachidonic acid levels, leading to migraines and other neurological manifestations, including fatigue, nausea, and brain fog [134]. Once again, CGRP seems to be one of the molecules most involved, both through vasodilation of the dural blood vessels and through an induced degranulation of the mast cells, resulting in the extravasation of plasma proteins underlying the neurogenic inflammation and pain of migraines [99,131].

## 6. Role of Anti-Allergic Drugs in Headaches

Several studies have investigated the efficacy of prophylactic pharmacologic and non-pharmacologic therapy in the migraine patient [135,136,137,138]. However, in recent decades, few studies have been conducted to demonstrate the efficacy of antihistamine drugs in managing patients who suffer from migraines [139]. Studies on the H1 and H2 antihistamines have shown contradictory results on the efficacy of these drugs in the prophylaxis of the migraine patient [57,135,140,141,142,143,144,145,146,147,148,149]. Some studies focusing on H3R [126,150,151,152,153] and H4R [129,154,155] have obtained more promising results [129,150,151,152,153,154,155,156]. Table 2 summarizes the antihistamines used for each specific receptor and whether the study conducted was a pre-clinical or clinical trial. The following paragraphs address these topics.

### 6.1. H1 and H2 Antihistamines

Studies of H1 and H2 antihistamines lack scientific strength and show contrasting results [140]. In a prospective double-blind clinical trial, the combination of nalbuphine (an opioid analgesic known to be effective in migraine) and hydroxyzine was not significantly more effective when compared with treatment groups administered with nalbuphine or hydroxyzine alone or compared with the placebo group [142]. Among H1 antagonists, cinnarizine and cyproheptadine have been reported to be effective in preventing migraines [55]. A significant relief in the frequency, duration, and severity of migraine attacks was recorded when cyproheptadine and propranolol (noncardioselective β blocker used in the prophylaxis of migraine) [158,159] were used independently [158,160] or in combination compared to a placebo [157]. One open-label pilot trial demonstrated a significant reduction in the number of migraine days and the intake of medication to treat acute attacks in patients treated with cinnarizine [143]. This evidence was confirmed by a subsequent open-label trial [57]. When the efficacies of cinnarizine and valproate were tested and compared, a significant decrease in terms of migraine symptoms was confirmed in both groups, although a greater efficacy of valproate was reported (55% vs. 36.4%) [144]. Experience in the adult population has suggested the use of these drugs in the prophylaxis of migraine in the pediatric population [140]: cinnarizine and sodium valproate were safe and effective for the prophylaxis of childhood patients, as indicated by two randomized double-blind placebo-controlled trials [145,146]. It is interesting to note that the chemical structure and the pharmacological profile of cinnarizine are similar to those of flunarizine [161], a “selective” calcium-entry blocker and histamine antagonist H1 receptor [161,162] known for its effectiveness in the prophylactic treatment of migraines in adults and children [163,164]. In contrast, in a study conducted in 1964, cyproheptadine was investigated in comparison with methysergide (a specific serotonin receptor antagonist) [165] and bellergal (a mixture of phenobarbitone, ergotamine tartrate, and belladonna alkaloids) [166], and no evidence of statistical improvement of migraine was demonstrated, with the placebo showing better outcomes [141]. A double-blind randomized placebo-controlled study evaluated the effect of clemastine (another H1 antihistamine) on migraine induction by pituitary adenylate cyclase-activating peptide-38 (PACAP38), albeit unsuccessfully [147]. Similarly, a histamine H2 blocker known as cimetidine, either alone or in combination with an H1 blocker (chlorpheniramine), was demonstrated to be ineffective in several studies [140,148,149]. In conclusion, H1 and H2 blockers have not definitively demonstrated an effective role in the prophylaxis of the migraine patient, and further high-quality randomized placebo-controlled studies in both children and adults are needed to confirm the data [55,140,141,142,143,144,146,157,167,168].

### 6.2. H3 and H4 Antihistamines

A feedback mechanism of histamine release [130,150,156,169] and the antinociceptive action [96,97,98,128,129,170] could offer a new therapeutic perspective in migraine prophylaxis [55,140]. However, until now, studies on H4R have only been conducted in animal models [129,154,155]. Interesting results have been reported in the studies conducted on n-alpha-methylhistamine [55,140], a histamine catabolite with a selective affinity for H3R [150,171,172]. Administration of a low dose of histamine or its catabolite might stimulate the H3R feedback loop, reducing histamine release [171]. In randomized, double-blind controlled trials, subcutaneous administration has proved the efficacy of n-alpha-methylhistamine in reducing the intensity, frequency, and duration of the headache [126,150,151,152,153]. Due to the inhibition of the neurogenic edema response involved in migraine pathophysiology [156], histaminergic H3R agonists reduce the intake of analgesic drugs in migraine patients [150], which is explained by the inhibition of plasma protein extravasation and the promotion of brain electrical activity, consistent with the different phases of the circadian rhythm [127]. Moreover, the authors proved that a low dose of histamine or subcutaneous n-alpha-methylhistamine has effects similar to, or even greater than, those of sodium valproate [126], topiramate [151], botulinum toxin type A [152], and propranolol [153]. Based on this evidence, other studies have attempted to evaluate the role of H4R (35–40% homology with H3R) [140]. Considering that amitriptyline, a well-known prophylactic drug for migraine [173], has shown affinity for H4R, there are positive expectations from molecules potentially capable of binding this receptor [174]. To clarify the pathophysiological role of this receptor, the H4R agonist “VUF 8430” has been tested in animal models via intracerebroventricular administration [129]. Neuronal H4R activation had an acute thermal analgesic effect, suggesting that H4R might be involved in the production of analgesia in the absence of an inflammatory process [129]. The same result has been achieved with the selective blockade of H4R instead of its agonist [154,155]. This evidence prevents a consensus on the impact of this receptor on migraine pathways. Further description of its localization and function is required [140]. In conclusion, H3 and H4 antihistamines appear promising in migraine prophylaxis [126,140,150,151,152,153,156], even if the actual data are still discordant [129,154,155].

## 7. Discussion

Migraines and allergic disorders are conditions with a high and increasing prevalence in the general population [12,14]. These conditions are associated with a socioeconomic burden and with a decrease in the quality of life, especially when both disorders coexist in the same individual [16]. Due to their incidence, these disorders may exist in the same patient even though they are not etiopathogenically related. However, several studies have highlighted that the bidirectional association between migraines and allergic disorders could be much more than a coincidence [17,18,19,20,21,22,23,24,25,26,27,28,29,30,31,32,33,34,35,36,37,38,39,40,41,42,43,59,60]. In particular, asthma, atopic dermatitis/allergic eczema, and allergic rhinitis are the allergic disorders most frequently reported in children, adolescents, and adults complaining of migraine. Although a clear epidemiological overlap has been established, it is not clear why some allergic disorders are more frequently reported than others. A possible explanation is that asthma, atopic dermatitis, and allergic rhinitis are the disorders most investigated as those associated with the greatest impact on patients’ quality of life and for which patients most often approach allergy doctors. The recurrence of migraine attacks and their severity depend on the interaction between genetic and environmental factors [69]. Any situation of malaise or discomfort can worsen the migraine in an individual who is genetically predisposed to them [76]. In this respect, patients with symptomatic allergic disorders are more likely to have attacks of high frequency and severity. Furthermore, the same environmental factors and emotional stress or psychological distress are associated with the occurrence or worsening of allergic conditions such as asthma and are related to the rate of emergency department attendance for migraine [175,176]. Immunological and vasoactive mediators, inflammation, and the involvement of parasympathetic and trigeminal nerves could play a combined role, which reinforces the common pathogenic link between these conditions. With these premises, it is difficult to exclude the possibility that the higher prevalence of migraines among the allergic population is simply due to a higher incidence of the disease. Although both disorders are frequently comorbid and may share common pathophysiological mediators and pathways, there is still not enough scientific evidence to draw a definitive conclusion regarding whether there is a direct relationship between migraines and allergic disorders. Both disorders undoubtedly remain two distinct conditions, each of which is genetically determined, but share some common pathogenic pathways. As summarized in this review, some evidence supports a role for histamine and the histaminergic system in several pathways associated with the pathophysiological mechanisms of migraines and allergic disorders. As a neurotransmitter of the CNS [92], histamine is involved in various brain activities that are often altered during a migraine, such as the sleep–wake cycle. Furthermore, playing a role in the immune system [94] and being a vasoactive mediator capable of modifying BBB permeability and neurogenic inflammation [127], histamine has been a much-studied therapeutic target in migraine prophylaxis. H1/H2R antagonists have produced contradictory results in the management of migraine patients [141,142,143,144,146,157,167]. For this reason, their use in clinical practice is far from recognized. Promising results have instead been obtained with substances that modulate H3/H4R [126,129,150,151,152,153,154,155,156]; however, many drugs have only been studied in the pre-clinical phase [140]. This study has several limitations: (1) The method of data collection in population-based studies (often through questionnaires or collection of patient data from pre-existing databases) may not have allowed the diagnosis of patients according to international definitions of migraine, asthma, allergic rhinitis, and other conditions. (2) In these studies, detailed information and laboratory data related to allergy, including serum IgE levels, eosinophil levels, allergic tests, and family histories of atopy, were unavailable. (3) Most studies have examined the correlation between migraines and isolated allergic disorders but not a combination of allergic disorders. (4) Basic studies have focused on describing the pathogenic pathways of migraine or allergic disorders, but there are none designed to examine common mechanisms between them. (5) The studies on the efficacy of antihistamine drugs in the prophylaxis of migraine have been conducted on small cohorts, which may limit the generalizability of the results; moreover, the studies have often not been randomized and have lacked comparison or placebo groups.

## 8. Conclusions

For centuries, clinical and epidemiological studies have recognized the overlap between migraines and allergic disorders. Whether it is a causal relationship is uncertain, and it cannot be excluded that migraines may merely be more recognizable in allergic patients since the malaise due to allergy worsens migraine symptoms. Though migraines and allergic disorders may share some common pathogenic pathways, it is far from clear whether there is a direct relationship between the two conditions. Whichever is the case, this review underlines the importance of considering allergic comorbid disorders among patients suffering from migraines so both conditions can be treated. Regarding future research directions, further studies are needed to gather more information on different aspects of this topic. Additional longitudinal studies in patients of all ages are required to verify the epidemiological overlap between migraines and allergic disorders. From the pathophysiological point of view, basic studies on the possible shared pathways (inflammatory, immunological, etc.) and systems (autonomic, histaminergic, etc.) for the development of new therapeutic strategies, are indicated. From the therapeutic point of view, pre-clinical studies and randomized, placebo-controlled clinical trials are needed to evaluate the preventive role of anti-allergic therapies in the occurrence of migraines in patients suffering from allergic disorders. From a clinical point of view, management of patients with migraines should also include screening for associated allergic symptoms. This could lead to a significant impact on public health due to the early identification and treatment possibilities of patients with comorbidities, improving their quality of life.

## Figures and Tables

**Table 1:** Epidemiology of the comorbidity of migraines and allergic disorders in population-based studies published since 2000. The summarized results column indicates whether there is an epidemiological association between these conditions, where “+” means there is an association and “−” means there is no association.

Reference	Type of Study	Age of the Target Group	Case, Exposed Group	Country of Study	Aim of the Study	Findings	Summarized Results
***Association between allergic/atopic disorders (including allergic rhinitis and atopic dermatitis), asthma, and migraines***							
**Sillanpää M et al., 2000** [17]	Prospective study	Children, 7 years, who were observed for 15 years	1205	Finland	To investigate headaches and their comorbidities	Both allergy and asthma were comorbid with the development of a headache (of any type).	+
**Özge A et al., 2006** [18]	Prospective cross-sectional clinical study	Adults, 18–46 years	186	Turkey	To explore the relationship between atopic disorders (asthma, allergic rhinitis (AR), conjunctivitis, seasonal allergy, food allergy, and drug allergy) and migraines	Of the patients suffering from migraines, 41.4% reported at least one atopic disorder.	+
**Lateef TM et al., 2012** [19]	Prospective survey study	Adolescents, 13–18 years	6843	US	To examine the pattern of and the extent to which other physical conditions are comorbid with migraines and other headaches	Asthma and seasonal allergies were more common in adolescents suffering from migraines than in adolescents with nonspecific headaches.	+
**Özge A et al., 2014** [20]	Prospective survey study	Children, 4–18 years	795	Turkey	To investigate atopic disorders in migraine patients	Atopic disorders (AR, conjunctivitis, and asthma) are statistically more commonly reported in patients suffering from migraine with aura (21.6%) than in those suffering from migraine without aura and tension-type headaches.	+
**Turan MO et al., 2017** [21]	Prospective study	Adults, 17–77 years	288	Turkey	To investigate the prevalence of migraines in asthma patients and to examine whether there is a relationship among atopic disorders, parental history, and migraines in asthma patients	There is a high prevalence of migraines in patients suffering from asthma. Patients with atopic symptoms were found to have a significantly high rate of headaches.	+
**Eidlitz-Markus T et al., 2017** [22]	Prospective survey study	Children, 2–18 years	401	Israel	To compare comorbidities between migraines and tension headaches in patients treated in a tertiary pediatric headache clinic	The main organic comorbidities of migraines and tension headaches were atopic disease, asthma, and first-reported iron-deficiency anemia.	+
**Graif Y et al., 2018** [23]	Retrospective study	Adolescents	113,671	Israel	To examine the association between specialist-diagnosed asthma and migraine among adolescents	Migraines were significantly more prevalent among adolescents with asthma than those without asthma. Multivariate analysis showed a significant association between migraine and both asthma and AR.	+
**Wei CC et al., 2018** [24]	Retrospective study	Children, 7–18 years	80,650	China	To investigate the interaction between children with antecedent allergic diseases and their future risks of migraine	Children with preceding allergic diseases (allergic conjunctivitis, AR, and asthma) had a greater subsequent risk of migraines than the controls.	+
**Buse DC et al., 2020** [25]	Prospective study	Adults, mean age 46 years	11,603 who participated in the American Migraine Prevalence and Prevention (AMPP) Study	US	To characterize the impact and burden of migraine in four monthly headache day categories	Allergies/hay fever were reported by 51.7%, asthma by 17.9%, and dermatitis/eczema endorsed by 6.6% of the sample of migraine patients. A comparison of high-frequency episodic migraine and low-/moderate-frequency episodic migraine found statistically significant differences in the rates of allergies (OR = 1.30) and asthma (OR = 1.34).	+
**Manjunath J et al., 2021** [26]	Retrospective study	Children	4898 who participated in the Fragile Families and Child Wellbeing Study	US	To understand the association of atopic dermatitis (AD) and comorbid asthma, sleep, and mental health disturbances with headaches	Children and adolescents with AD, particularly those with sleep disturbances and atopic and mental health comorbidities, had increased headaches.	+
**Han JH et al., 2023** [27]	Retrospective study	Adults, more than 20 years	3,607,599, using the Korean National Health Insurance Service Database	Korea	To investigate atopic disorders and the risk that they may lead to a migraine	Patients with atopic disorders (AD, AR, and asthma) may have an increased risk of migraine, and the larger the number of concomitant atopic disorders, the higher the risk of migraine.	+
***Association between asthma and migraine***							
**Davey G et al., 2002** [28]	Retrospective matched case–control study	Adults	129,356, using the General Practice Research Database (GPRD)	UK	To examine whether there is an association between migraine and asthma	There is an increased risk of asthma in migraine patients. Among definite migraine cases, the relative risks of chronic obstructive pulmonary disease, respiratory symptoms, eczema, and hay fever were raised.	+
**Ronchetti R et al., 2002** [59]	Retrospective cross-sectional survey	Children, 6–14 years	2472	Italy	To evaluate the extra-respiratory symptoms (such as headaches) associated with asthma	Headache was not significantly associated with asthma.	−
**Aamodt AH et al., 2007** [29]	Prospective cross-sectional study	Adults, more than 20 years	51,383 who participated in Head-Nord-Trøndelag Health Study (HUNT)	Norway	To examine the relationship between migraine and no-migraine headaches and asthma	Both migraine and no-migraine headaches were approximately 1.5 times more likely among those with current asthma and asthma-related symptoms than those without.	+
**Becker C et al., 2008** [60]	Retrospective study	Adults	103,376	UK	To estimate the risk of asthma in patients with a general practitioner-diagnosed migraine	The risk of developing asthma was not materially altered for patients with a migraine diagnosis.	−
**Lateef TM et al., 2009** [30]	Retrospective study	Children, 4–18 years	10,198 who participated in the National Health and Nutrition Examination Surveys	US	To determine the comorbidity of recurrent headaches in children in the United States	Asthma was found to be more common in children suffering from headaches, with at least one of these occurring in 41.6% of the children suffering from headaches versus 25.0% of children free from headaches.	+
**Fernandez-de-las-penas C et al., 2010** [31]	Prospective survey study	Adolescents and adults, more than 16 years	29,478 who participated in Spanish National Health Survey	Spain	To estimate the prevalence and comorbidities of migraine	Subjects suffering from migraines were significantly more likely to have comorbid self-reported asthma.	+
**Karlstad Ø et al., 2011** [32]	Prospective cross-sectional study	Children and adults	37,060	Norway	To examine the occurrence of chronic diseases in an asthma population compared to that in a non-asthmatic control group	There was an increased prevalence of migraines in asthmatics compared with the control group.	+
**Czerwinski S et al., 2012** [33]	Prospective study	Adults	3731	US	To evaluate the association between migraine and asthma and to estimate the risk of hypertensive disorders of pregnancy in relation to maternal comorbid migraine and asthma	Migraineurs had 1.38-fold increased odds of asthma compared with non-migraineurs. The odds of hypertensive disorders of pregnancy were the highest among women with comorbid migraine and asthma.	+
**Martin VT et al., 2016** [34]	Prospective study	Adults, mean age of 50.4 years	4446 who participated in American Migraine Prevalence and Prevention Study	US	To test the hypothesis that, in persons with episodic migraine, asthma is a risk factor for the onset of chronic migraine	Asthma is associated with an increased risk of new-onset chronic migraine.	+
**Peng YH et al., 2018** [35]	Retrospective study	Adults, 20–60 years	33,235 using the National Health Insurance Research Database	China	To examine whether adult migraine patients are at a higher risk of developing asthma	The hazard ratio of asthma development was 1.37 for the migraine group compared with the nonmigraine group after adjustment for age, sex, occupational status, insurance premium, urbanization, comorbidities, and annual outpatient department visits.	+
**Kim SY et al., 2019** [36]	Retrospective study	Adults	376,338 using the Korean Health Insurance Review and Assessment Service	Korea	To evaluate the bidirectional association between asthma and migraines using control subjects matched by demographic factors	Asthma and migraines are reciprocally significantly associated.	+
***Association between allergic/atopic disorders and migraine***							
**Ku M et al., 2006** [37]	Prospective survey study	Children and adults, 5–81 years	294	US	To determine the prevalence of migraines in AR patients	There is a high prevalence of migraines in patients with AR compared with those without AR.	+
**Artto V et al., 2006** [38]	Prospective survey study	Adults, mean age: 40.0 years	1000 who participated in the Finnish Migraine Gene Project	Finland	To investigate the comorbidity of migraine in migraine families	Migraine patients reported significantly more allergies compared to their family members without migraine.	+
**Martin VT et al., 2011** [39]	Prospective study	Adults, 18–65 years	536	US	To determine whether the degree of allergic sensitization and the administration of immunotherapy are associated with the prevalence and frequency of migraines in patients with allergic rhinitis	Lower “degrees of atopy” are associated with less frequent migraine attacks in younger adults, while higher degrees are associated with more frequent migraines.	+
***Association between atopic dermatitis/eczema and migraine***							
**Saunes M et al., 2007** [40]	Prospective survey study	Adolescents, 13–19 years	8817 who participated in the Young-HUNT study	Norway	To study self-reported mental distress among patients with AD compared with healthy adolescents and mental distress among those with other chronic diseases (e.g., headaches)	Migraines and atopic disorders are frequently comorbid, with the frequency of 15.8% in girls and 7.1% in boys.	+
**Silverberg JI. 2016** [41]	Prospective cross-sectional study	Children and adolescents	401,002 who participated in the National Survey of Children’s Health	US	To determine whether eczema is associated with increased headaches	Eczema is significantly associated with increased headaches, particularly in patients with severe disease accompanied by atopy, fatigue, and sleep disturbances.	+
**Shreberk-Hassidim R et al., 2017** [42]	Prospective study	Adolescents	1,187,757	Israel	To estimate AD prevalence, trends, and association with demographic factors and comorbidities	There was a significantly higher prevalence of migraines in patients with AD.	+
**Fan R et al., 2023** [43]	Retrospective study	Adults	214,866	US	To investigate the association between AD and migraine	Patients with AD had a significantly higher rate of migraine diagnosis than the non-AD cohort.	+

**Table 2:** Antihistamine drugs in migraine prophylaxis. In the Findings column, “+−” means comparable effective, “−” means less effective, “− −” means markedly less effective, “+” refers to greater effectiveness, and “++” means much more effective.

Target	Type	Matching	Findings	Reference
H1	CYPROHEPTADINE (antagonist)	vs. methysergide	− −	[141] (1964)
		vs. bellergal	+	[141] (1964)
		vs. placebo	++	[141] (1964)
	CYPROHEPTADINE (antagonist) + Propranolol	vs. cyproheptadine	+	[157] (2000)
		vs. propranolol	+	[157] (2000)
		vs. placebo	++	[157] (2000)
	HYDROXYZINE (antagonist) + Nalbuphine	vs. placebo	− −	[142] (1987)
	CINNARIZINE (antagonist)	vs. baseline symptoms	++	[143] (2003)
		vs. baseline symptoms	++	[57] (2006)
		vs. valproate	−	[144] (2013)
		vs. placebo	++	[145] (2014)
		vs. valproate	+	[146] (2020)
		vs. placebo	++	[146] (2020)
	CLEMASTINE (antagonist)	Control of PACAP38-induced migraine vs. placebo	− −	[147] (2019)
H2	CIMETIDINE (antagonist)	vs. placebo	− −	[148] (1978)
	CIMETIDINE (antagonist) + Chlorpheniramine	vs. placebo	− −	[148] (1978)
	CIMETIDINE (antagonist)	vs. placebo	− −	[149] (1980)
	CIMETIDINE (antagonist) + Chlorpheniramine	vs. placebo	− −	[149] (1980)
H3	NALPHA-METHYLHISTAMINE SC (agonist with low doses)	vs. placebo	++	[150] (2003)
	NALPHA-METHYLHISTAMINE SC titrate up (agonist with low doses)	vs. placebo	++	[156] (2006)
	NALPHA-METHYLHISTAMINE SC titrate up (agonist with low doses)	vs. propranolol	+−	[153] (2014)
	HISTAMINE SC titrate up (agonist with low doses)	vs. valproate	+−	[126] (2007)
	HISTAMINE SC titrate up (agonist with low doses)	vs. topiramate	+−	[151] (2008)
	HISTAMINE SC titrate up (agonist with low doses)	vs. botox	+−	[152] (2009)
H4	VUF 4830 (agonist)	nociception induction (animal models)	++	[129] (2013)
	JNJ7777120 (antagonist)	nociception induction (animal models)	++	[155] (2007)
	JNJ7777120 (antagonist)	nociception induction (animal models)	++	[154] (2009)
	VUF 6002 (antagonist)	nociception induction (animal models)	++	[155] (2007)

## Data Availability

Not applicable.
